# Efficacy of Stem Cell Application in Diabetes Mellitus: Promising Future Therapy for Diabetes and Its Complications

**DOI:** 10.7759/cureus.13563

**Published:** 2021-02-26

**Authors:** Faten R Bani Hamad, Neelam Rahat, Kavya Shankar, Nicholas Tsouklidis

**Affiliations:** 1 Medicine, California Institute of Behavioral Neurosciences & Psychology, Fairfield, USA; 2 Neurology, California Institute of Behavioral Neurosciences & Psychology, Fairfield, USA; 3 Health Care Administration, University of Cincinnati Health, Cincinnati, USA; 4 Medicine, Atlantic University School of Medicine, Gros Islet, LCA

**Keywords:** diabetes mellitus, t1dm, t2dm, stem cell, bm-hsc, bm-mnc, msc, ucb, stem cell transplantation, stem cell therapy

## Abstract

Diabetes mellitus (DM) is a serious and common chronic disease with high morbidity and mortality rates. Recently, stem cell-based therapy has shown considerable promise as a future therapeutic modality for DM. This review aims to summarize the types of stem cells that have the most successful evidence in treating type 1 diabetes mellitus (T1DM) and type 2 diabetes mellitus (T2DM), and also to assess the safety and efficacy of different types of stem cells in the treatment of DM. PubMed, MEDLINE, and PubMed Central databases were searched up to October 15, 2020, using medical subject heading (MeSH) terms. After application of inclusion criteria and exclusion criteria, 10 studies were included in our final review: six T1DM studies including 120 patients, and four T2DM studies including 65 patients. Our data showed that autologous and allogeneic stem cell therapy is a relatively safe and effective method for selected individuals with DM. The best therapeutic outcome was achieved by transplantation of bone marrow hemopoietic stem cells (BM-HSCs) for T1DM and bone marrow mononuclear cells (BM-MNCs) along with mesenchymal stromal cells (MSCs) for T2DM. However, patients with DKA are not a good candidate for stem cell transplantation. Further rigorous experiments are needed in order to be able to establish stem cell-based therapies as the future standard of care for treating DM.

## Introduction and background

Diabetes mellitus (DM) is one of the fastest-growing international health problems of the 21st century [1]. According to a report from the International Diabetes Federation, there were 463 million diabetic patients worldwide in 2019. This number is expected to grow to 538 million by 2030 [1]. Diabetes mellitus types 1 DM (T1DM) and type 2 DM (T2DM) are chronic diseases that occur when blood glucose levels are elevated [1]. T1DM is caused by autoimmune destruction of insulin-producing beta-cells of the pancreas, resulting in partial or complete insulin deficiency. The cause of this reaction is not fully understood; however, it is believed to be due to a combination of genetic predispositions and environmental triggers that initiate the destructive autoimmune process [2,3]. The pathophysiological mechanism in T2DM includes insulin resistance with beta-cell dysfunction [4]. DM can result in multi-systemic chronic complications, particularly microvascular diseases (i.e., retinopathy, nephropathy, and neuropathy) and macro-vascular diseases (i.e., cardiovascular diseases, cerebrovascular accidents, and peripheral vascular diseases), with high morbidity and mortality rates [1,5]. The use of therapeutic exogenous insulin and other hypoglycemic agents have been employed to control hyperglycemia; however, they delay but don't prevent the occurrence of DM-related complications and fail to mimic the secretion of endogenous insulin which may cause unstable glucose levels, reactive hypoglycemia, and disruption to the patient's work and life in general [5]. Stem cell therapy, islet cell transplantation, pancreas transplantation, and anti-CD3 monoclonal antibodies, as an alternative modality to cure DM, have been approved for clinical use for both T1DM and T2DM [5-8]. The Collaborative Islet Transplant Registry (CITR) demonstrates that 44% of recipients were insulin-independent for three years post-transplantation, and the development of DM complications has been successfully postponed. However, islet cell transplantation has multiple drawbacks, including the limited supplies of donors and their high variability [6]. According to United Network for Organ Sharing (UNOS) data registry analysis, pancreatic transplantation successfully obtains insulin independence in 60% of recipients for four years after transplantation. Drawbacks include the high morbidity associated with major surgery (70% survival after seven years), limited availability of donor pancreas, and lifelong immunosuppression and its risks-- infection and malignancy [7]. On the other hand, anti-CD3 monoclonal antibody therapy, although considered moderately safe, obtained insulin independence in only 5% of participants after two years of follow-up [8].

Stem cells represent a highly promising new modality of treatment for DM and diabetic complications owing to their immunomodulatory properties, multilineage differentiation, and regeneration potential [5,9]. Multiple types of stem cells have been tested for their ability to regenerate functional insulin-producing cells-- early or embryonic stem cells, induced pluripotent stem cells, and mature or adult stem cells, for example, umbilical cord blood stem cells (UCB), peripheral blood mononuclear cells (PB-MNCs) and bone marrow mononuclear cells (BM-MNCs), which mainly contain bone marrow mesenchymal stromal cells (BM-MSCs) and bone marrow hemopoietic stem cells (BM-HSCs). Pancreatic and hepatic stem cells have also been tested [9]. Bhansali et al. used bone marrow-derived stem cells to treat T2DM patients and reported encouraging results [10]. Voltarelli et al. showed that hematopoietic stem cells (HSCs) are also a safe and effective treatment for newly diagnosed T1DM patients to improve β-cell function [11]. On the other hand, Giannopoulou et al. have shown that the application of cord blood in children with type 1 diabetes is safe, but fails to preserve beta-cell function [12]. However, many concerns still require further research surrounding types of stem cells used to ensure effectiveness in the treatment of DM, the transplantation procedure, and long-term recovery [13,14].

This review article aims to summarize the types of stem cells that have the most successful evidence in treating T1DM and T2DM, and also to assess the safety and efficacy of different types of stem cells in the treatment of DM. Safety can be defined as the absence of adverse side effects and efficacy as an enhancement in pancreatic islet beta cells function after therapy, which can be indexed by improvements in laboratory parameters such as HbA1c, C-peptide level, fasting plasma glucose (FPG), and insulin requirements.

## Review

Methods

Search Strategy

A systematic search of studies published from inception until October 15, 2020, was performed in PubMed, MEDLINE, and PubMed Central databases. To accurately identify clinical trials of stem cell transplantation in DM patients, a search was performed using a combination of medical subject heading (MeSH) terms and text words: "diabetes mellitus, type 1" or "diabetes mellitus, type 2" and "stem cell transplantation" and "therapy" or "therapeutic use." 

Inclusion and Exclusion Criteria

English language restrictions were applied, articles in languages other than English were selected only if the English translation was available. Filters were set for human studies and clinical trials. The inclusion criteria were studies with the following elements: (1) patients diagnosed with T1DM or T2DM; (2) stem cell therapy with no more than one type of stem cell transplanted in specific study participants; (3) minimal follow-up period for at least six months after the initiation of the therapy; (4) studies in which the participants had no altered endocrine status other than DM; (5) availability of laboratory parameters for diabetes, for example, HbA1c, C-peptide, FPG, and insulin requirements. Exclusion criteria were studies on animals, reviews, or studies for which the full text was unavailable. To ensure the quality of the included studies, we did not include gray literature. All studies were peer-reviewed. 

Study Selection

Electronic search yielded a total of 478 citations. After removal of duplication, irrelevant studies, animal trials, molecular studies, reviews, and abstract only articles, 25 studies remain to assess for eligibility. After application of the inclusion and exclusion criteria, 10 studies were included in our final review.

Results

Characteristics of the Included Studies

After the selection process, 10 eligible clinical trials with a total of 185 patients were included in the present review [10-12,15-21]. The characteristics of the 10 included studies are presented in Table 1. The studies were published from 2007 to 2017 and represented an international population, as they were performed in a large range of countries, including China, Brazil, India, Sweden, and Germany. Stem cell therapy was evaluated in patients with either T1DM (six studies, 120 patients, mean age 17.7 years) or T2DM (four studies, 65 patients, mean age 51.4 years). Considering the type and source of cells, three studies used BM-HSCs (T1DM, 56 patients) [11,15,16], two studies used BM-MNCs (T2DM, 30 patients) [10,17], four studies used MSCs (T1DM, two studies, 47 patients; T2DM, two studies, 42 patients) [17-20], and two studies used UCB (T1DM, one study, 17 patients; T2DM, one study, three patients) [12,21]. Among them, one study compared the efficacy of BM-MSCs and BM-MNCs in T2DM therapy [17]. It is noteworthy that Only five studies included a control group, which received either placebo or insulin, with the therapy group receiving stem cell transplantation [12,15,17,19,20]. The follow-up period ranged from six months to 29.8 months.

**Table 1:** Characteristics of the Included Studies. T1DM: type 1 diabetes mellitus; T2DM: type 2 diabetes mellitus; BM-HSCs: bone marrow hematopoietic stem cells; BM-MNCs: bone marrow mononuclear cells; MSCs: mesenchymal stem cells; UCB: umbilical cord blood; NA: not available; IV: intravenous; m: months

Regimen	Author And year	Country	Sample type	Sample size (cell therapy/ control)	Mean age (cell therapy /control)	Mean dose of injected cells	Mode of injection	Mean follow-up period
BM-HSC	Ye et al., 2017 [15]	China	T1DM	8 /10	18.86 /20.18	NA	IV	12 m
	Couri et al., 2009 [16]	Brazil	T1DM	23	18.4	10.52 × l0^6^/kg	IV	29.8 m
	Voltarelli et al., 2007 [11]	Brazil	T1DM	15	19.2	11 × l0^6^/kg	IV	18.8 m
BM-MNC	Bhansali et al., 2017 [17]	India	T2DM	10 /10	44.5 /53.5	1 ×10^9^/kg	Superior pancreaticoduodenal artery	12 m
	Bhansali et al., 2009 [10]	India	T2DM	10	57.5	3.5 × 10^8^/kg	Superior pancreaticoduodenal artery	6 m
MSC	Bhansali et al., 2017 [17]	India	T2DM	10 /10	50.2 /53.5	1 × 10^6^/kg	Superior pancreaticoduodenal artery	12 m
	Liu et al., 2014 [18]	China	T2DM	22	52.9	1 × 10^6^/kg	IV on Day 5+ splenic artery on Day 10	12 m
	Carlsson et al., 2014 [19]	Sweden	T1DM	9 /9	24 /27	2.75 × l0^6^/kg	IV	12 m
	Hu et al., 2013 [20]	China	T1DM	15 /14	17.6 /18.2	2.6 × 10^7^/kg	IV	21 m
UCB	Giannopoulou et al., 2013 [12]	Germany	T1DM	7/ 10	3.02/ 6.6	1.27 × 10^6^/kg	IV	12 m
	Tong et al., 2013 [21]	China	T2DM	3	41	2.88 × 10^6^/kg	Intrapancreatic	6 m

Discussion

**Figure 1:**
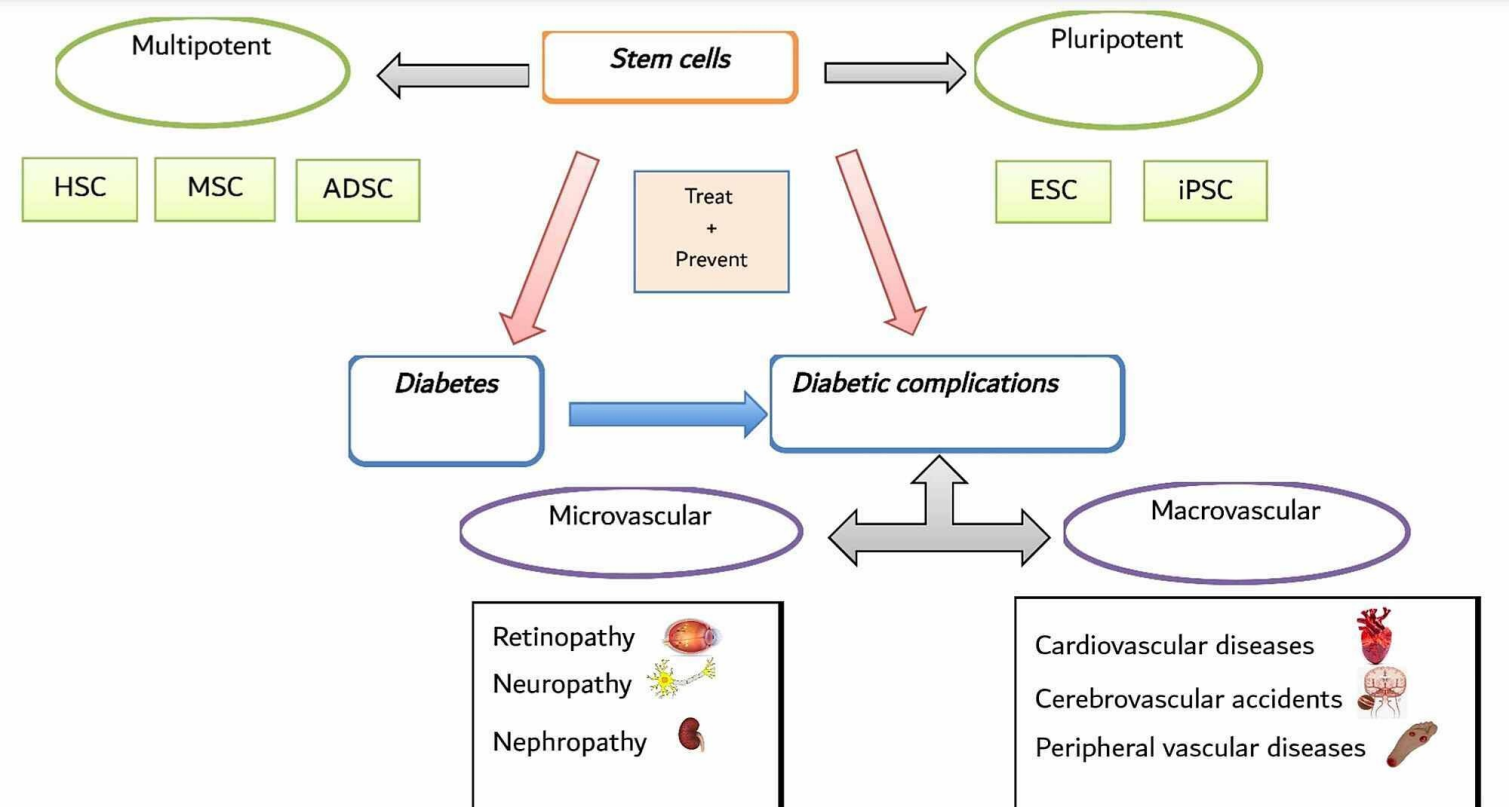
Overview of Stem Cell Therapy in Diabetes and Its Related Complications. HSC: hematopoietic stem cells; MSC: mesenchymal stromal cells; ADSC: adipose-derived stem cells; ESC: embryonic stem cells; iPSC: induced pluripotent stem cells

DM with its highly growing rates is a serious threat, which needs to be addressed cautiously [1]. Traditional modalities of therapy are focused on regulating the glucose level but don't prevent the occurrence of diabetic complications [5]. Cell-based therapy, such as stem cells, which are unspecialized cells that evolve into specialized cells with a multilineage differentiation and regeneration potential, which varies to different tissue types, is considered as a new promising therapeutic modality for DM. Multiple studies have proved the various impacts, safety, and efficacy of both pluripotent and multipotent stem cells in treating DM and its related complications (Figure 1) [5,9].

Limitations

The major limitation of this paper is the small number of studies that were included. Furthermore, there were multiple limitations in our involved studies that require larger, randomized, double-blinded, and better-controlled experiments with longer follow-up period future studies. Additionally, comparative studies are needed to decide the stem cell type, number, and transplantation method that produces the best result for human patients, as well as further studies that address the precise factors and pathways involved in cell homing, microenvironment improvement, and interactions between stem cells and islet progenitor cells are needed in order to be able to establish the stem cell-based therapies as the standard of care for treating DM.

The question remains, with all these modifications in future experiments, could stem cell-based therapy be the novel therapy and the end of DM?

Treatment of diabetes type 1 in clinical trials with humans

BM-HSC

In Brazil, in 2007, the first-ever clinical trial to evaluate the safety and efficacy of stem cell transplantation in T1DM was done by Voltarelli and his colleagues. An autologous non-myeloablative hematopoietic stem cell (AHST), with a mean dose of 11 ×l0^6^/kg, was followed by the immunosuppressive therapy IV injected in 15 newly diagnosed T1DM patients (mean age 19.2 years). Patients with previous diabetic ketoacidosis (DKA) were excluded after the first patient with DKA failed to improve after AHST [11]. During seven to 36 months of follow-up (mean 18.8 m), 14 patients experienced variable periods of insulin independence; the vast majority of them maintained this status throughout the follow-up period, the mean total C-peptide values, which indicated the endogenous insulin secretion, were significantly greater than the pre-treatment values after six months of treatment and were stabilized up to 24 months after treatment, HbA1c levels were sustained below 7% in 13 patients, and Anti-glutamic acid decarboxylase (anti-GAD) antibody levels decreased after six months of treatment, and were maintained up to 24 months thereafter. However, one patient suffered pneumonia, and two patients developed late endocrine dysfunction (hypothyroidism or hypogonadism). Additionally, the majority of patients showed symptoms of neutropenia, alopecia, nausea, and vomiting as an adverse effect of immunosuppressants. There was no mortality [11]. The study showed multiple limitations, including a non-randomized design, absence of a control group, small sample size, and short follow-up period. However, these results are encouraging and support AHST as a promising therapy for T1DM [11].

After two years, to investigate if the results in the previous study were due to preservation of beta-cell mass continued monitoring was performed after stem cell transplantation by Couri et al. in the same research laboratory of the previous study in the 15 original and eight additional patients (mean age 18.4 years), the mean dose of IV injected CD34 cells was 10.52 x10^6^/kg [16]. Patients with DKA were excluded as in the 2007 study [11]. During seven to 58 months of follow-up (mean 29.8 m), 20 patients became insulin-independent, 12 continuously and eight transiently, for periods as long as four years associated with good glycemic control. In the continuous insulin-independent group, C peptide levels increased significantly up to 24 months and were sustained until at least 36 months post-transplantation, and HbA1c levels were less than 7%. In the transient insulin-independent group, C peptide levels increased significantly up to 36 months and were sustained until at least 48 months post-transplantation; however, oligospermia was the most frequent complication noticed in nine patients. Moreover, two patients developed bilateral nosocomial pneumonia, and three patients developed late endocrine dysfunction. There was no mortality [16]. This study added additional proof of AHST efficacy in treating T1DM and also demonstrated that these results are due to improvement in beta-cell function after AHST. Like the 2007 study, there were limitations like non-randomized design, absence of a control group, and small sample size [16].

In 2017, a study was conducted in China to explore the immune response after AHST in T1DM. 18 newly diagnosed T1DM patients were enrolled, eight patients received AHST (therapy group, mean age 18.86 years), and 10 patients received insulin-only therapy (control group, 20.18 years) [15]. After 12 months follow-up, the AHST group showed a significant reduction in T-helper type 1 (Th1) and T-helper type 17 (Th17) cell proportions and function, which play an important role in the inflammatory and autoimmune destructive process, downregulation of their cytokines expression (IFN-γ, IL-2, IL-12p40, and IL-17A) as well as their transcription factors (Tbet and ROR-rt). The proportion of regulatory (Treg) cells, which play an important role in regulating immunity, was decreased in the control group but remained at similar levels in the therapy group, the final result was more Treg cells and higher Treg associated cytokines levels (IL-10, TGF-β, and foxp3) in the AHST group than the Insulin- only group. Also, the patients in the AHST group showed better residual C-peptide secretion, lower anti-GAD titers, and less exogenous insulin dosages compared with the insulin-only group [15]. This study showed that AHST leads to improve islet beta cells function and reset of immunity, which means a new and more tolerant immune system is generated after transplantation, which leads to weakening of autoimmune inflammatory reactions, halt further insulin-producing cells destruction, and decelerate diabetes progression. There are two main limitations in this study including small sample size and a short follow-up period [15].

MSC

In China, in 2013, Hu et al. used Wharton's jelly-derived mesenchymal stem cells (WJ-MSCs) from the umbilical cord in newly diagnosed T1DM. In this randomized controlled clinical trial, 29 patients were enrolled, 15 patients received a mean of 2.6 ×10^7^/kg IV injected WJ-MSCs treatment (therapy group, mean age 17.6 years), and 14 patients received normal saline, which is similar to the parenteral solution of WJ-MSCs in the appearance and volume (control group, mean age 18.2 years), both groups also treated with intensive insulin therapy before and during the 21 months follow-up period [20]. During the follow-up period, patients in the therapy group showed significant improvement in C peptide and HbA1c levels. Mean postprandial plasma glucose (PPG) levels were more controlled with less fluctuation compared with patients in the control group, the dosage of exogenous insulin was significantly reduced and three out of 15 patients become insulin-independent in the therapy group. There were no adverse events [20]. WJ-MSCs lead to improve islet beta cells function and showed safety and efficacy as a treatment for T1DM. The main limitation in this study was the small sample size [20].

Two years later, in Sweden, another randomized controlled clinical trial added Additional proof for the safety and efficacy of MSC as a treatment for T1DM. Eighteen newly diagnosed T1DM patients were enrolled and divided into two groups; the therapy group (nine patients, mean age 24 years, treated with a mean of 2.75 ×l0^6^/kg IV injected autologous BM-MSC), and the control group (nine patients, mean age 27 years, treated only with insulin) [19]. During one-year of follow-up, the C- peptide concentrations in blood in response to a mixed-meal tolerance test (MMTT) were the major difference between the groups. In the therapy group, the C-peptide response was preserved or even increased. The control group showed a decrease and eventually loss of C-peptide values and peaks. There were no side effects [19]. The major limitations in this study including a small sample size and a short follow-up period [19]. 

UCB

In Germany, in 2013, a non-randomized controlled trial was done to evaluate the safety and efficacy of using autologous UCB in newly diagnosed T1DM children. Seventeen patients were enrolled and divided into two groups: therapy group with seven patients (mean age 3.02 years) received 1.27 × 10^6^/kg IV autologous UCB therapy, and a control group with 10 patients (mean age 6.6 years) received insulin- only therapy [12]. During 12 months follow-up period, there were no significant changes in metabolic (C-peptide, HbA1c, and insulin requirement) and immune (autoantibodies titter, and T-cell response) parameters between the therapy and control group. However, the C-peptide values showed significantly better outcomes with more infused CD34+ cells dose. No adverse side effects were observed [12]. The study showed that the UCB is safe but not effective in treating T1DM. The main limitations including small sample size, non-randomized design, and signiﬁcant age difference between the two groups [12]. 

Treatment of diabetes type 2 in clinical trials with humans

BM-MNC

In India, in 2009, a study was conducted to assess the safety and efficacy of autologous BM-derived stem cell transplantation in T2DM using a mean dose of 3.5 × 10^8^/kg intrapancreatic infusion of autologous BM-MNC, which retrieved by aspiration from the posterior iliac crest. Ten T2DM patients (mean age 57.5 years) with triple oral anti-diabetic drug failure and insulin-dependence were enrolled [10]. During six months of follow-up, insulin requirements were reduced significantly and three patients become insulin-independent, reduction in HbA1c, improvement in C-peptide levels, and significant weight loss also have been observed. There were no serious adverse events [10]. This study indicated that BM-MNC is safe, effective and leads to improvement of beta-cell function in T2DM. It also showed that a higher infusion dose would have been more effective and correlated with the reduction of insulin requirements. The main limitations including non-randomized design, absence of a control group, small sample size, and short follow-up period [10].

MSC

Liu et al. showed the safety and efficiency of allogeneic WJ-MSC in T2DM indicated by enhancement of beta-cell function and immunological regulation after transplantation. The study was conducted on 22 T2DM patients (mean age 52.9 years) in China in 2014. All patients received WJ-MSC with the dose of 1x10^6^/kg twice, via IV injection on day five the first time and via splenic artery using catheter directly injected to pancreas on day 10 in the second time [18]. During 12 months of follow up, there were an elevation in C-peptide values and reduction in HbA1c, blood glucose levels, insulin, and oral anti-diabetic drugs doses, and systemic immunological and inflammatory markers, which include: T- lymphocytes counts (CD3+, CD4,+ and CD8+ cells) and pro-inflammatory cytokines (IL-6, IL-10, IL-1,β, and TNF-α). There were no significant adverse side effects [18]. The main limitations including a small sample size, non-randomized design, absence of a control group, and short follow-up period [18].

In 2017, a study was conducted to compare the safety and efficacy of using autologous BM-MNCs and BM-MSCs in T2DM. Thirty patients on triple oral anti-diabetic drugs with insulin were selected for participation and divided into three groups, 20 patients involved in two therapy groups, therapy group (1) in which 10 patients received a dose of 1x10^9^/kg BM-MNCs, and therapy group (2) in which 10 patients received 1x10^6^/kg of BM-MSCs. Both BM-MNCs and BM-MSCs were retrieved by aspiration from the posterior iliac crest and then infused direction to the pancreas. The remaining 10 patients who received placebo were involved in the control group [17]. During 12 months of follow-up, there was a significant reduction of more than 50% in exogenous insulin requirements in both therapy groups, a significant increase in stimulated C-peptide values in therapy group (1), and significant improvement of insulin sensitivity along with increase expression of IRS-1 gene in therapy group (2), six out of 10 patients who received MSCs showed a significant Wight loss. There were no significant adverse side effects [17]. This study gave more evidence on the safety and efficacy of MSCs and MNCs transplantation in T2DM. Furthermore, it's suggested that combining both MNC and MSC could lead to better outcomes and glycemic control. The limitations including a small sample size and a short follow-up period [17].

UCB

In a clinical trial conducted in China in 2013, three patients with T2DM (mean age 41 years) were enrolled and received a mean of 2.88 × 10^6^/kg UCB cells infused intra-pancreatic via a dorsal pancreatic artery. After transplantation and during the follow-up duration of six months, the C- peptide levels increased, insulin requirements decreased, and more stabilization in blood glucose readings after UCB infusion than before was observed in all patients. No transplantation-related adverse side effects were observed [21]. These results indicated the effectiveness and safety of UCB transplantation in T2DM. The main limitations including small sample size, non- randomization, the absence of a control group, and a short period of follow-up [21]. 

Our data show that the best therapeutic outcome for T1DM was achieved with CD34+ HSC therapy, which showed significant improvements in DM laboratory parameters like C-peptide and HbA1c levels and endogenous insulin secretion. For MSC transplantation, Carlsson et al. reported no significant changes in HbA1c, C-peptide levels, or amount of insulin usage after BM-MSCs transplantation [19]. Hu et al. reported that both the HbA1c and C-peptide levels in UC-MSC group patients were significantly better than both pre-therapy values and those in control group patients during the follow-up period [20]. Therefore, UC-MSCs appear to be more effective than BM-MSCs in the treatment of T1DM. However, there are few studies on MSC treatment for T1DM. More studies are needed to explore which source of MSCs is more favorable for treatment [14]. In conclusion, we can consider that treatment with MSCs is as safe and effective but not significant as treatment with HSCs, which makes BM-HSCs superior to MSCs in the treatment of T1DM. While UCB therapy, despite it can be administered safely to children with T1DM without adverse side effects, showed the poorest outcome as it failed to improve C-peptide, AbA1c, and insulin requirements. In T2DM, MSCs whether derived from bone marrow or umbilical cord and BM-MNCs transplantation, showed favorable therapeutic effects with significant outcomes. Unlike T1DM, UCB therapy showed effectiveness in T2DM.

Adverse side effects that were observed in both T1DM and T2DM stem cell recipients have mostly resulted from the administration of high-dose immunosuppression. However, almost all these side effects were resolved shortly after therapy, and there was no mortality reported. This makes stem cell therapy much safer compared with whole organ and islet transplantation. Better-tolerated, lower-dose immunosuppressive regimen along with using anti-inflammatory agents and stronger prophylaxis against infection paired with autologous stem cell transplantation may lead to better outcomes [22,23]. Other important factors that affected the treatment outcome included: (1) the time of DM diagnosis, patients who received therapy earlier after diagnosis with DM were more likely to achieve insulin independence than those with later diagnosis, this is possibly related to decreased capacity of beta-cell reservoir over time which impedes therapeutic efficacy of stem cells, and this is also can explain why patients with DKA are not a good candidate for stem cell transplantation [24]; (2) number of injected cells (<10^7^ or >=10^7^) was positively correlated with C-peptide and HbA1c levels and reduced doses of insulin requirements [25]; (3) method of cells delivery (IV or intrapancreatic administration), intrapancreatic infusion showed better improving in DM lab parameters than IV infusion [26]; and (4) autologous transplantation, autologous treated groups showed better response to treatment than allogeneic treated groups due to decrease risk of immune rejection in autologous transplantation [23].

## Conclusions

Stem cells are undifferentiated cells that play a critical role in the evolution and rebirth of soft tissues and body growth. During the previous years, many experiments utilized stem cells solely or in conjunction with other healing methods and revealed the effectiveness and safety of stem cells in a variety of illnesses such as diabetes. The best therapeutic outcome was achieved by transplantation of BM-HSCs for T1DM and BM-MNCs along with MSCs for T2DM. However, patients with DKA are not a good candidate for stem cell transplantation.
